# General Hospitals and Infectious Cases

**Published:** 1895-01-26

**Authors:** 


### EDITOR'S LETTER-BOX.

[Our correspondents are reminded that prolixity is a great bar to publication, and that brevity of style and conciseness of statement greatly facilitate early insertion.]

### "GENERAL HOSPITALS AND INFECTIOUS CASES."

SIR,—You have fallen into a serious but, I am sure, an unintentional error in the paragraph under the above heading, which appears in THE HOSPITAL for January 19th, 1895, page 284. There is not one medico-chirurgical hospital in Dublin which admits fever (with the exception of typhoid) and infectious cases into its general wards. It is not true that "the Dublin hospitals have been always nearly a century behind other hospitals of the world with regard to this question." On the contrary, either "fever and infectious cases" are absolutely denied admission—as is the case at Mercers', St. Vincent's, Jervis Street, and (at present) the Mater Misericordiæ Hospitals—or such cases are treated in specially-constructed, isolated, epidemic hospitals standing on the grounds of the general hospitals, but in every way carefully kept separate from the main buildings This is the plan adopted at the City of Dublin, the Adelaide, the Meath, the House of Industry, Sir Patrick Dun's, and Steevens' Hospitals. The Meath Hospital, of which I am senior physician, is at present building a new fever wing, with thirty beds, at a cost of £7,000; and every precaution is being taken to secure perfect safety against infection. Small-pox, I should add, is jealously excluded from even the epidemic wings of the general hospitals because of its great striking distance. Trusting that you will insert this reclamation in a spirit of fair play, I am, Sir, yours faithfully, JOHN WILLIAM MOORE.

40, Fitzwilliam Square West, Dublin, January 19th, 1895.

*** The statement referred to was based upon information from an official source to the effect that infectious cases are still treated in the general hospitals of Dublin. We saw this system at work in Dublin hospitals when we inspected them some years ago, and Dr. Moore states that such cases are still treated by the staff of at least six Dublin hospitals, though the patients are housed in separate wings. Having regard to the site difficulties attending general hospitals in towns, and to general hygienic considerations, we are of opinion that it is not desirable or even right to treat infectious cases in any but separate hospitals situated on isolated sites where every precaution can be taken to prevent any risk of infection to outsiders. Sir Patrick Dun's Hospital is an old offender, as Dr. Moore must know; and we much regret to hear of the expenditure of so large a sum as £7,000 upon a fever wing to the Meath Hospital.—ED. *T. H.*

